# Impact of exposure to conflict, tsunami and mental disorders on school absenteeism: findings from a national sample of Sri Lankan children aged 12–17 years

**DOI:** 10.1186/1471-2458-13-560

**Published:** 2013-06-08

**Authors:** Chesmal Siriwardhana, Gayani Pannala, Sisira Siribaddana, Athula Sumathipala, Robert Stewart

**Affiliations:** 1 Health Service & Population Research Department, Institute of Psychiatry, King's College London, UK; 2 Institute for Research & Development, Colombo, Sri Lanka; 3 Department of Medicine, Faculty of Medicine & Allied Sciences, Rajarata University of Sri Lanka, Mihintale, Sri Lanka

## Abstract

**Background:**

Armed conflicts and natural disasters are common. Millions of people, including children are killed, injured, disabled and displaced as a result. The effects of conflict and natural disaster on mental health, especially of children are well established but effects on education have received less attention. This study investigated associations between conflict and/or tsunami exposure in Sri Lanka and their associations with absenteeism in a national sample of school children.

**Methods:**

A cross-sectional survey was conducted in 2006–7 among 1,505 randomly selected school children aged 12–17 years attending government schools in 17 districts. The hypotheses were that absenteeism would be more common in children previously affected by conflict or the 2004 tsunami and that at least part of this effect would be accounted for by mental disorders. Survey information included socio-demographic, conflict and tsunami exposure, mental health status (Strengths and Difficulties Questionnaire) and information on absenteeism (defined as 20% or greater non-attendance over one year).

**Results:**

The total sample of consisted of 1,505 students aged 12–17 years with a mean age of 13.7 years. 120 children reported at least one conflict exposure and 65 reported at least one tsunami exposure while only 15 reported exposure to both conflict and tsunami. Prevalence of emotional disorder caseness was 2.7%, conduct disorder caseness 5.8%, hyperactivity disorder caseness 0.6%, and 8.5% were identified as having any psychiatric disorder. Absenteeism was present in 26.8%. Overall, previous exposure to tsunami (OR 2.29 95% CI 1.36-3.84) was significantly associated with absenteeism whereas exposure to conflict was not (OR 1.32 95% CI 0.88-1.97), although some specific conflict-related exposures were significant risk factors. Mental disorder was strongly associated with absenteeism but did not account for its association with tsunami or conflict exposure.

**Conclusions:**

Exposure to traumatic events may have a detrimental effect on subsequent school attendance. This may give rise to perpetuating socioeconomic inequality and needs further research to inform policy and intervention.

## Background

Armed conflicts and recurring natural disasters are common and the damaging effects are especially salient in developing countries, compounded by poverty. Exposure to these events can create lasting detrimental effects on physical and mental health of both adult and child populations. The effects of exposure to conflict and natural disaster on health, especially mental health, are well established [1-4] and international studies report high prevalence of mental disorders among children and adolescents exposed to conflicts and natural disasters [5-9].

The longer term impact of traumatic events may be compounded by disruptions to education, not only directly because of the event itself but also in the longer term through its impact on behavior. Since a person’s opportunities later in life depend strongly on their educational attainment and experiences, this is a major issue for child populations affected by traumatic events [10]. Absenteeism refers to excusable or inexcusable absences from primary/secondary school system [11]. There is homogeneity in global research findings on absenteeism and related behavior [11,12]. An array of psychiatric co-morbidities, such as depression, anxiety, oppositional defiant disorder, conduct disorder and behavioral disorder has been found to precipitate and exacerbate absenteeism but the role of mental disorder in the relationship between traumatic events and school attendance is less well studied [11,13-17].

Since achieving independence in 1948, Sri Lanka has achieved relatively higher success compared to its regional neighbors in both health and educational sectors [18,19]. Sri Lanka has near-universal, gender-equitable access to primary education, with approximately 4 million students in 10,000 schools along with a primary school net enrollment rate of 99.4% for males and 99.9% for females [19-22]. However, over the last 35 years Sri Lanka has witnessed many natural and man-made disasters including two armed conflicts in the south, a civil conflict in the north and the tsunami disaster in 2004 [23,24]. The adverse impact of these exposures on child mental health has received some limited investigation, but impacts on education have not been considered [8,10,25-27]. Utilizing data from a large survey of school children in Sri Lanka, we sought to investigate the associations of absenteeism with previous conflict and tsunami exposure and to investigate the role of mental disorder in these associations.

## Methods

### Setting: the Sri Lankan National Mental Health Survey (NMHS)

This epidemiological survey was carried out by the Institute for Research & Development (IRD) in 2006/2007, commissioned by the Sri Lankan Ministry of Health and funded by the Health Sector Development Project, World Bank as the first island-wide attempt to generate up-to-date and accurate information on prevalence, etiology and impact of mental disorders in the population in order to inform healthcare planning. A school survey was built into this project, the main objectives of which were to estimate the prevalence of conduct and behavioral disorders among school children, describing associated demographic and socioeconomic characteristics, potential social and environmental risk factors. This survey was conducted about three years after the 2004 tsunami and in the midst of a civil conflict which had been ongoing for three decades.

### Sample: NMHS schools survey study design

The schools-based component of the NMHS was originally designed as a cross-sectional survey to be conducted among school children aged between 6–17 years from all 25 administrative districts in Sri Lanka. However, due to the active civil conflict at the time, it could only be conducted in 17 districts (although many of the sampled districts had been affected by the conflict). As the majority of student population (around 96%) attends the state school education system, the survey focused on this sector. State schools are categorized into four main types (type 1AB-up to grade 13 with Advanced Level science, arts and commerce subject streams, type 1C-up to grade 13 with Advanced Level arts and commerce streams, type 2-up to grade 11 and Ordinary Level, type 4-up to grade 5 and primary) for administrative purposes, based on the availability of grades and subject streams and reflecting the age, socio-economic background and resources available at schools. The school-attending age group in the primary and secondary education system is between 6–19 years. However, school children aged over 17 years were excluded due to the uncertain validity of the Strengths and Difficulties Questionnaire (SDQ) in this age group. This age group also overlapped with the inclusion criteria of the community adult component of the NMHS. The heterogeneous nature of school children, schools and administrative structures was taken into account in sample size calculation. Accordingly, the sample was stratified into children at primary stage (grade 1–5; 5–10 year olds), and at junior secondary stage of education (grade 6–11; 11–17 year olds). The school was considered as the primary sampling unit and individual children as the secondary sample unit (in clusters of 30 students per school), selected in two stages utilizing school attendance registers [28]. Up to two additional visits were undertaken in instances of the randomly selected student being unavailable (absent) during the initial visit. However, no replacements were included and no children were interviewed at their homes. The analyses described here were restricted to participants aged 12 and over who had completed questions on the exposures of interest (exposure to conflict and tsunami). Data from the 6–11 age group were insufficiently complete, as the questionnaires of interest had been predominantly administered to the older age group, and participants in the younger age range were not considered in the analyses presented here.

Ethical approval was obtained from the Ethics Review Committee of the Faculty of Medical Sciences, University of Sri Jayawardenepura, Sri Lanka. Informed written consent was obtained from children, their parents and teachers where necessary.

### Hypotheses and conceptual framework

**Figure 1:**
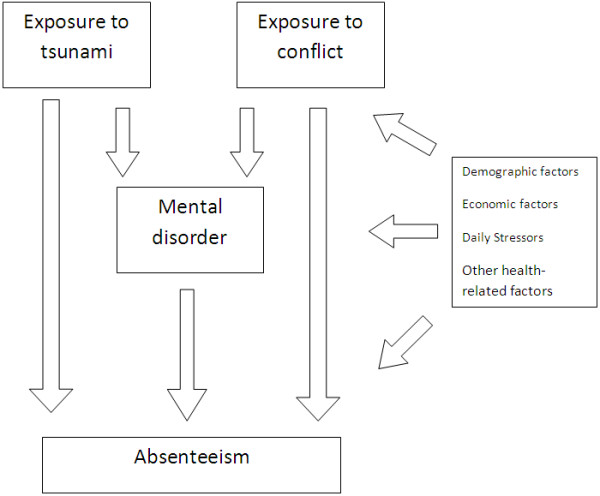
Conceptual framework-Exposure to conflict, tsunami and mental disorder and association with school absenteeism.

The key research question for this paper based on the national data was to explore the impact of exposure to conflict, tsunami on school absenteeism in Sri Lanka and the role of mental disorders in accounting for this. It was broadly hypothesized that children affected by conflict/disasters would have a higher tendency to develop absenteeism due to the variety of factors (social, cultural, health-related, economic, daily stressors) associated with these exposures, and that at least part of this association might be accounted for by mental disorders. Therefore, four separate hypotheses were developed: 1.) Exposure to war and related events is associated with increased absenteeism; 2.) Exposure to tsunami and related events is associated with increased absenteeism; 3.) Exposure to mental disorders is associated with increased absenteeism; 4.) Exposure to conflict and/or tsunami related event is associated with increased mental disorders. The conceptual framework diagram (Figure 1) shows the hypothesized relationships between the analysed variables.

### Assessments

Data collection was carried out by research assistants (teachers, master teachers and government education officials) trained on official survey procedures, selection and randomization of respondents, obtaining informed consent and other ethical issues, as well as in the administration of study instruments. The interviewing teachers were not from the same schools selected for the study. Data entry was conducted using SPSS statistical software [29] by trained data entry operators. A variety of instruments considered valid for research in low and middle income settings were used. These had already been translated and back translated in to the main languages (Sinhalese & Tamil) of the country. Data was collected from the children, parents and teachers as each instrument required (no reply alternatives were used for the SDQ). Of the data collected, the following measures were considered in the analysis presented here:

Socio-demographic information: age, sex, ethnicity (Sinhala, Tamil, Muslim), area of residence (urban, suburban, rural), type of school (as categorized in the methodology).

Family circumstances (and proxy measure of socio-economic status): maternal and paternal level of education.

Student mental health: the full multi-informant Strengths and Difficulties Questionnaire (SDQ) was administered. This instrument is designed to enquire about 25 items on psychological attributes divided into 5 sub-scales of 5 items to measure conduct problems, hyperactivity-inattention, emotional symptoms, peer problems, and pro-social behavior of children between 4–17 years [30,31]. A total difficulties score (1–40) is generated by summing four problem sub-scale scores (conduct, hyperactivity, emotional and peer problems) which excludes the pro-social scale. Using the SDQ predictive computerized algorithm [32], final outcome scores can be classified as normal, borderline, and abnormal categories to identify likely cases with mental disorders (conduct, hyperactivity, emotional and any psychiatric disorder). The computerized algorithm has been developed to predict child psychiatric illnesses on the basis of the symptom and impact scores derived from SDQ versions completed by parents, teachers and children [32]. SDQ prediction by the algorithm matching an independent clinical diagnosis has been shown to be substantial and highly significant (Kendall's tau-b 0.49-0.73; p < 0.001) with "abnormal" SDQ prediction for any given disorder correctly identifying 81-91% of the children with a confirmed clinical diagnosis, showing that the algorithm is sufficiently accurate and robust to be of practical value [32]. Although the scores can also be used as continuous variables, for the purpose of analysis in this paper, algorithm-based SDQ outcome was used as dichotomous (normal/abnormal) with borderline and abnormal categories combined. The SDQ has been used as a research tool locally and internationally to assess the prevalence of psychiatric morbidity among children [33-35].

School attendance: absenteeism was the primary outcome for this analysis, recorded by using the percentage of total days absent from the total days of schooling available over a year to students and applying a cutoff point of 20% in order to define the presence or not of absenteeism as a binary variable. This information was recorded by the research assistants for each participant student by accessing class attendance registers available at schools.

Tsunami and conflict exposure: these were the primary independent variables for this analysis and were measured by using short questionnaires with eight items for each exposure which had predominantly been administered to participants aged 12 years and over. These items were; direct participation in conflict/being in an affected area during tsunami, direct injury due to conflict/tsunami, loss (death) of close family or close friends due to conflict/tsunami, injury of close family or close friends due to conflict/tsunami, loss (death) of other family or friends due to conflict/tsunami, injury of other family or friends due to conflict/tsunami, loss of property due to conflict/tsunami, displacement due to conflict/tsunami. One or more positive answers out of 8 in the separate questionnaires were used to define ‘exposure’ for conflict and tsunami and these participants were compared against the remainder for primary analyses. For illustrative purposes, secondary analyses additionally described relationships between each individual exposure and absenteeism, where cell sizes were sufficient.

### Statistical analysis

Data was weighted to account for the school student population variations. STATA statistical software was used for data analyses [36]. Initial analyses described demographic characteristics and frequency of the exposure and outcome variables. Unadjusted analyses investigated associations between primary exposures (any conflict exposure, any tsunami exposure, mental disorder) and outcome (absenteeism), followed by exploratory analyses of individual conflict/tsunami exposures and absenteeism where cell sizes allowed. Finally unadjusted analyses investigated the associations between conflict/tsunami exposure and the SDQ mental disorder categories.

Logistic regression analyses were then carried out to investigate the independence of associations of interest. In exploring the associations between exposure to conflict and tsunami with absenteeism, three separate models were included: model 1 (socio-demographic variables-age, gender, ethnicity, area of residence and type of school), model 2 (model 1 + proxy socio-economic status variables-paternal and maternal education), model 3 (model 2 + mental disorder). Mental disorder outcome associations with absenteeism were explored through 4 separate models; model 1 (socio-demographic variables-age, gender, ethnicity, area of residence and type of school), model 2 (model 1 + paternal and maternal education), model 3 (model 2 + conflict exposure), model 4 (model 2 + tsunami exposure).

## Results

### Sample characteristics

Overall, the school survey had a participation rate of 92.5%. The total sample of secondary age group (12–17 years) which was considered in the analysis for this paper consisted of 1,505 participants with the mean age of 13.7 years (SD 1.3); 729 male and 757 female. Further distributions of demographic covariates are summarized in Table 1. Of the total sample, 120 (36.2%) children reported at least one conflict exposure and 65 (49.1%) reported at least one tsunami exposure. Only 15 (46.1%) reported exposure to both conflict and tsunami. A total of 127 (8.5%) students were identified as having any psychiatric disorder. Prevalence of emotional disorder caseness was 2.7%, conduct disorder caseness 5.8% and hyperactivity disorder caseness 0.6%.

**Table 1:** Socio-demographic covariates, conflict, tsunami and mental disorder exposures with absenteeism

**Characteristic**	**Number in group N = 1505**	**Absenteeism% N = 404**	**Unadjusted OR (95% CI)**
**Gender**			
Female	729	26.9	Reference
Male	757	34.8	**0.69 (0.54-0.87)**
**Age**			
12–14	1027	69.7	Reference
15–17	478	30.0	0.98 (0.76-1.27)
**Ethnicity**			
Sinhala	1316	29.1	Reference
Tamil	153	50.4	**2.47 (1.66-3.65)**
Muslim	33	17.2	0.50 (0.19-1.33)
**Type of school**			
Type 1AB	134	12.6	Reference
Type 1C	640	30.1	**2.99 (1.58-5.62)**
Type 2	693	34.0	**3.56 (1.90-6.67)**
Type 3	38	28.9	**2.81 (1.11-7.11)**
**Maternal education**			
Less than grade 6	533	29.3	**1.78 (1.21-2.61)**
Grade 6 to O/L	598	28.9	**2.75 (1.87-4.05)**
More than O/L	262	18.5	Reference
**Paternal education**			
Less than grade 6	541	37.3	**2.17 (1.39-3.37)**
Grade 6 to O/L	556	28.7	**3.20 (2.06-4.96)**
More than O/L	226	15.6	Reference
**Area of residence**			
Rural	1,270	29.1	Reference
Suburban/urban	79	33.8	1.24 (0.74-2.06)
Coastal/plantations	135	46.5	**2.11 (1.40-3.19)**
**Main caregiver**			
Mother/Father	1,407	31.3	Reference
Others	90	21.3	0.59 (0.33-1.04)
**Conflict exposure**			
No exposure	1,327	30.1	Reference
Any exposure	120	36.2	1.32 (0.88-1.97)
**Tsunami exposure**			
No exposure	1,369	29.6	Reference
Any exposure	65	49.1	**2.29 (1.36-3.84)**
**Conflict and tsunami exposure**			
Exposure to conflict and tsunami	15	46.1	-
**Mental disorder**			
No disorder	1,352	28.6	Reference
Any psychiatric disorder	127	49.1	**2.40 (1.63-3.55)**
Conduct disorder	87	48.6	**2.29 (1.43-3.65)**
Emotional disorder	41	51.2	**2.48 (1.30-4.70)**
Hyperactivity disorder	10	44.4	1.83 (0.49-6.87)

*CI* Confidence Interval, *OR* Odds Ratio.

Note: Absenteeism was calculated as the total number of days absent from the total number of available school days with a cut-off at 20%.

Note: Statistically significant odds ratios are highlighted.

### Prevalence and distribution of absenteeism

A total of 404 (26.8%) students met the criterion determining absenteeism. Among them, males, younger students (aged 12–14 years), type 2 school attending students, students from coastal/plantation areas and those with lower parental education reported higher rates of absenteeism (Table 1). Absenteeism was present in 49.1% of students reporting tsunami exposure and in 36.2% of those reporting conflict exposure. Students with emotional disorders had the highest prevalence of absenteeism (51.2%) while those in conduct disorder (48.6%), any psychiatric disorder (49.1%) and hyperactivity disorder (44.4%) were roughly similar.

### Unadjusted associations between trauma and absenteeism

The unadjusted association between any conflict exposure and absenteeism was positive but not significant (OR 1.32 95% CI 0.88-1.97) while that with any tsunami exposure was statistically significant (OR 2.29 95% CI 1.36-3.84) as shown in Table 1. Additional exploratory analyses of individual conflict exposures (displayed in Additional file 1: Table S1) showed that absenteeism was significantly associated with reported injury of a close family member, with displacement and with loss of property (p-values < 0.05) as well as being in participants who reported sustaining injury to self. Of individual tsunami exposures, absenteeism was significantly associated with loss (death) of friends or other family (p < 0.05), as well as being present in all participants who reported sustaining injury or loss of a close family member.

### Associations of mental disorders with absenteeism and trauma exposures

Significant unadjusted associations were also found between absenteeism and the following SDQ categories (Table 1): any psychiatric disorder (OR 2.40; 95%CI 1.63-3.55), conduct disorder (OR 2.29; 95%CI 1.43-3.65) and emotional disorder (OR 2.48; 95%CI 1.30-4.70). However, no significant associations were found between exposure to conflict and any psychiatric disorder (OR 1.20; 95%CI 0.64-2.25), conduct disorder (OR 1.15; 95%CI 0.54-2.45) or emotional disorder (OR 1.23; 95%CI 0.43-3.25). Similarly, no significant associations were found between exposure to tsunami and any psychiatric disorder (OR 1.09; 95%CI 0.46-2.59), conduct disorder (OR 1.34; 95%CI 0.52-3.43) or emotional disorder (OR 1.17; 95%CI 0.27-4.98).

### Adjusted analyses

In the logistic regression analyses summarized in Table 2, the association between any conflict exposure and absenteeism remained weak and non-significant, while the association with any tsunami exposure remained significant after adjustment and relatively unaltered in strength in all models. In further logistic regression analyses displayed in Table 3, associations of absenteeism with any psychiatric disorder, conduct disorder and emotional disorder remained significant in all models after adjustment (Table 3). The association between absenteeism and emotional disorder strengthened after the adjustments (in models 3 and 4) while the association between absenteeism and conduct disorder weakened (in models 3 and 4). Adjustment for paternal/maternal education attenuated the strength of association for all four mental disorder outcomes.

**Table 2:** Association of exposure to conflict and tsunami with absenteeism

**Association with absenteeism**		
**Covariate models**	**Conflict exposure **^**a**^	**Tsunami exposure **^**b**^
	**OR (95% CI)**	**OR (95% CI)**
Unadjusted	1.32 (0 .88-1.97)	2.29 (1.36-3.84)
Model 1^c^	1.27 (0 .83-1.93)	2.03 (1.18-3.48)
Model 2^d^	1.39 (0.89-2.18)	1.93 (1.08-3.46)
Model 3^e^	1.29 (0.84-1.96)	2.05 (1.19-3.53)

*CI* Confidence Interval, *OR* Odds Ratio.

^a^ Exposure to at least one conflict related event.

^a^ Exposure to at least one tsunami related event.

^c^ Socio-demographic variables (age, gender, ethnicity, area of residence, type of school).

^d^ Socio-demographic + Socio-economic variables (paternal and maternal education).

^e^ Socio-demographic + Socio-economic variables + Any mental disorder.

**Table 3:** Adjusted associations of mental disorder outcomes with absenteeism

**Covariates in model**	**Any psych. disorder **^**a**^	**Conduct disorder **^**b**^	**Emotional disorder **^**c**^	**Hyperactivity disorder **^**d**^
	**OR (95% CI)**	**OR (95% CI)**	**OR (95% CI)**	**OR (95% CI)**
Unadjusted	2.40(1.63-3.55)	2.29(1.43-3.65)	2.48(1.30-4.70)	1.83(0.49-6.87)
Model 1^e^	2.27 (1.52-3.39)	2.01 (1.24-3.26)	2.48 (1.28-4.80)	1.81(0.44-7.31)
Model 2^f^	1.79 (1.14-2.82)	1.54 (0.88-2.70)	2.05 (1.00-4.19)	0.89(0.18-4.22)
Model 3^g^	2.29 (1.52-3.44)	1.99 (1.22-3.25)	2.60 (1.33-5.09)	2.31(0.52-10.24)
Model 4^h^	2.32(1.53-3.50)	1.87 (1.14-3.07)	3.00 (1.50-6.00)	2.36(0.53-10.48)

*CI* Confidence Interval, *OR* Odds ratio.

^a, b, c, d^ Final outcomes obtained through the SDQ algorithm.

^e^ Socio-demographic variables (age, gender, ethnicity, area of residence, type of school).

^f^ Socio-demographic + Socio-economic variables (paternal and maternal education).

^g^ Socio-demographic + Socio-economic variables + Conflict exposure.

^h^ Socio-demographic + Socio-economic variables + Tsunami exposure.

## Discussion

This study focused on two key traumatic exposures and their associations with absenteeism in a sample of Sri Lankan school children aged 12–17 years. The findings from this study are important because these types of exposure have received relatively little attention with respect to their later impact on children’s education despite the importance that this has in determining later social mobility and socioeconomic status. Key findings were that overall previous tsunami-related exposure was significantly associated with absenteeism, overall conflict-related exposure was not significantly associated with absenteeism, although some associations were found with individual conflict-related exposures in secondary analyses. Mental disorders were associated with absenteeism but did not appear to account substantially for the association between tsunami exposure and this outcome.

There is no consensus definition of significant absenteeism to our knowledge, and it varies according to the cut-off points applied by different authors [11,12,37,38]. Although numerical definition of absenteeism varies, the broad concept of absenteeism has been consistent, defined as the total number of absent days from school from the total number of available school days of a calendar year [11,37,38]. In our study, absenteeism, defined as non-attendance on at least 20% of school days, as recorded by the school, was present in 26.8% of the sample. This is generally consistent with the 20% absenteeism rate found in other studies in Sri Lanka and elsewhere [11,37-39].

This study was conducted in mid 2007, approximately two and half years after the 2004 December tsunami. In contrast, the civil conflict had been ongoing for almost three decades at the time of the survey. These timeframes have to be considered in interpreting the results for the two exposures of interest. A previous study on exposures to tsunami and conflict were both shown to be associated with mental ill health among Sri Lankan children [26]. Another study found a range of tsunami-associated psychosocial events such as loss of parents, prolonged displacement, losses of other family or friends to be associated with psychiatric symptomatology including depression and PTSD among Sri Lankan children [40]. However, our findings did not show any strong association between exposure to tsunami and mental disorders. Mental disorder therefore did not appear to be an important factor in our analyses accounting for the tsunami-absenteeism association. However, the impact of tsunami exposure on absenteeism may be due to the more disruptive, sudden impact of the exposure on social structures in the Sri Lankan context, and it is noteworthy that bereavement was the strongest individual tsunami-associated risk factor for absenteeism. Although exposure to conflict may be devastating, families tend to group together even in displacement, which might have limited the strength of association below significance for this overall exposure. The tsunami on the other hand may have disrupted whole social support systems, breaking families, communities and destroying alternative support systems such as schools within a very short span of time. In exploratory analyses, displacement and loss of property due to conflict were associated with absenteeism, which may support this suggestion of social disruption as an important underlying factor. In interpreting the findings from this study, attention must be paid to the fact that absenteeism may be linked more to the loss of family and its continuing effects (possibly exacerbated by more widespread social disruption), rather than to a direct link with the disaster in question.

A study carried out in eastern Sri Lanka found that daily stressors partially mediated the relation between exposure to conflict or disaster and psychological outcomes among adolescents [10]. The association with absenteeism in our sample remained relatively independent of most covariates, with paternal/maternal education level (proxy measure of socio-economic status) appearing to be the only major confounding factor. Furthermore, no significant associations were found between exposure to conflict and mental disorders in this study. Exposure to conflict can bring devastating changes to lives of children, especially if they are subjected to mass displacement and separations, leading to disruptions in their daily lives, most importantly to their education [2,9]. In the Sri Lankan setting, several districts included in the study bordered the conflict areas and people were subjected to displacement because of conflict-related violence spilling over, prior to and at the time of survey. This may be reflected in absenteeism of children in these populations [25,27] and the significant associations with conflict-related family injury, displacement and property loss in our sample.

Conflict and natural disaster related events can be highly traumatic to children with both short and long term consequences, affecting their physical and mental performance, and education is a key facet of their lives with a high vulnerability to disturbance, especially as education is perceived as the gateway for escaping poverty and attaining upward social mobility in most resource-poor settings in the world. The Strengths and Difficulties Questionnaire used in the study gave rise to an overall prevalence of 8.5% for any psychiatric disorder among the population of children in the sample. This figure is similar to findings on behavioral disorders (using similar outcome measures) among children in Sri Lanka and other countries [41]. Conduct disorders had the highest prevalence in the studied sample. Associations were observed for SDQ-derived conduct and emotional disorder with absenteeism. These findings add to existing evidence that mental illness such as behavioral disorders in children have a detrimental effect in their education, inducing and increasing absenteeism among them [11,16,17]. Regression analysis also showed a significant association between absenteeism and any psychiatric disorder that lies within the predictive range of the SDQ. This includes conduct, emotional and hyperactive disorders in a broader aspect. This evidence again suggests that psychopathology may play a key role in children’s education.

This study has certain limitations. The results may be subject to type II statistical error due to lack of power-for example, the relatively small numbers with specific conflict- or tsunami-related exposures which precluded detailed analyses of individual exposures. Type I errors are unlikely because of the primary focus on two exposures and one outcome; however, secondary associations need to be treated with more caution. The main likely biases are selection bias stemming from under-sampling of absentee students and information bias due to the data collection strategy used in the study. The fact that sampling was not possible in the districts most strongly and recently affected by conflict may have diluted the influence of this exposure, as well as limiting national generalisability. Under-sampling of absentee students, which may have lead to an increase of students without absenteeism being recruited to the study has been potentially minimized by conducting three visits to track selected participants in case they were absent while the overall participation rate was high at 92.5%. Another issue is that the analysis is limited to the secondary age students (12–17 years) and the findings do not reflect mental disorder prevalence of the younger age school children nor their school attendance patterns. Paternal education level was the only possible confounder shown in the regression analysis, although illnesses, stress among family members and daily stressors (not tested in the study) may act as confounders. As this was a cross-sectional study, causality underlying associations cannot be assumed.

## Conclusion

This paper presents evidence that exposure to certain natural or man-made traumatic events and mental disorders can have a detrimental effect on school attendance. Although every child has a right to education, many children are deprived on this basic right, especially in regions affected by the aftermath of conflict or disaster. Along with this deprivation, absenteeism due to mental and physical illness together with socio-economic factors reduces the chance of children receiving a proper education.

Evidence presented in this paper strengthens the call for expanding research into the impact of exposure to conflict and/or disasters on general educational attainment of children, especially in resource-poor settings. In particular, factors such as female gender and residence in a coastal/plantation area maybe predictors of absenteeism. This evidence is important for both local and wider policies directed towards achieving universal education, gender equality, reducing the impact of mental ill health, and improving social mobility. These findings may be utilized by international bodies, government agencies, non-governmental organizations and other stakeholders in managing educational, health and other related issues. In considering the needs of children, their education and mental health, these findings contribute to the evidence base for policy formulation and implementation. This paper also presents national data on school absenteeism in Sri Lanka, which can be used to inform policy to reduce inequalities across the national education system, especially for the secondary age group students. Service provision by government and other agencies also stands to be improved. There is a need to investigate further the effects of mental ill-health and related stressors on absenteeism and overall educational performance in children-for example the longer term impact on achievements and socio-economic status (and the ability of children to catch up after earlier disadvantage). Such studies will undoubtedly improve understanding of the complex associations in these fields of enquiry. Researchers, both in wider mental health and education fields, can usefully formulate research questions to address important gaps in evidence.

## Competing interests

The authors declare that they have no conflicts of interests.

## Authors’ contributions

CS conceived the paper, participated in the coordination and data collection of the main study. CS also performed secondary data analysis and wrote the first draft of the paper. AS and SS conceived the main study and participated in the data collection and management. GP managed the data and performed the initial data analysis. RS advised on the paper and edited the second draft of the paper. All authors read, edited and approved the final draft.

## Pre-publication history

The pre-publication history for this paper can be accessed here:

http://www.biomedcentral.com/1471-2458/13/560/prepub

## Supplementary Material

Additional file 1: Table S1 Associations between individual conflict/tsunami exposures and absenteeism. Click here for file
