# Supplementary material for: Impact of exposure to conflict, tsunami and mental disorders on school absenteeism: findings from a national sample of Sri Lankan children aged 12–17 years
Source: BMC Public Health. 2013 Jun 8;13:560. doi: 10.1186/1471-2458-13-560 (PMC3698150; doi:10.1186/1471-2458-13-560)
Supplement: Additional file 1: Table S1 — Associations between individual conflict/tsunami exposures and absenteeism. [file 1471-2458-13-560-S1.docx]

Additional file 1

Table S1 - Associations between individual conflict/tsunami exposures and absenteeism

| **Individual conflict/Tsunami exposures** | **Number exposed** | **Absenteeism** | **Unadjusted**  **OR (95% CI)** |
| --- | --- | --- | --- |
| **Conflict** | | | |
| Direct participation | 0 | 0 | * |
| Sustain injury | 2 | 2 | * |
| Lost (death) close family | 12 | 4 | 1.12 (0.33-3.75) |
| Close family injured | 24 | 12 | **2.28 (1.01-5.13)** |
| Lost (death) friend or other family | 51 | 19 | 1.35 (0.75-2.42) |
| Friend or other family injured | 43 | 12 | 0.83 (0.42-1.64) |
| Displaced | 24 | 16 | **3.70 (1.66-8.23)** |
| Lost property | 10 | 9 | **9.17 (1.94-43.42)** |
| **Tsunami** | | | |
| In an affected area | 21 | 21 | * |
| Sustain injury | 3 | 3 | * |
| Lost (death) close family | 1 | 1 | * |
| Close family injured | 8 | 4 | 2.26 (0.56-9.08) |
| Lost (death) friend or other family | 34 | 19 | **2.94 (1.47-5.85)** |
| Friend or other family injured | 20 | 6 | 0.96 (0.36-2.52) |
| Displaced | 6 | 2 | 1.12 (0.20-6.16) |
| Lost property | 3 | 2 | 4.51(0.40-49.91) |

*Insufficient cell sizes/failure of regression model
